# Association between obesity, quality of life, physical activity and health service utilization in primary care patients with osteoarthritis

**DOI:** 10.1186/1479-5868-5-4

**Published:** 2008-01-28

**Authors:** Thomas Rosemann, Richard Grol, Katja Herman, Michel Wensing, Joachim Szecsenyi

**Affiliations:** 1 Department of General Practice, University of Zürich, Rämistr.100, 8001 Zürich, Switzerland; 2 Department of General Practice and Health Services Research, University Hospital Heidelberg, Voßstr. 2, 69115 Heidelberg, Germany; 3 Radboud University Nijmegen Medical Centre, 6500 HB Nijmegen, The Netherlands

## Abstract

**Objective:**

To assess the association of obesity with quality of life, health service utilization and physical activity in a large sample of primary care patients with osteoarthritis (OA).

**Methods:**

Data were retrieved from the PraxArt project, representing a cohort of 1021 primary care patients with OA. In 978 patients, height and weight were measured and the Body Mass Index (BMI) was calculated. The AIMS2-SF was used to assess quality of life (QoL). Data about health service utilization (HSU) were retrieved by means of patients' medical files. Concomitant depression was assessed by means of the Patient Health Questionnaire (PHQ-9). Patients were grouped into normal weight, overweight and obese according to the definition of the WHO and compared by means of analysis of covariance (ANCOVA).

**Results:**

Obese and overweight persons achieved significantly higher scores on the AIMS2-SF lower body scale, the symptom, the affect and the work scale, indicating an increased burden by OA. The PHQ-9 score increased significantly over the three weight-groups, indicating a positive association of BMI and depression. With increasing BMI, the number of comorbidities increased and physical activity decreased significantly. After controlling for covariates, contacts to orthopaedics and performed x-rays remained significantly higher in obese patients, but not contacts to general practitioners.

**Conclusion:**

The results display a strong association of QoL and BMI, resulting in increased use of the health care system. Thus, the study emphasizes the need for appropriate approaches in primary care to break the vicious circle of overweight, depression, decreasing physical inactivity and decreasing QoL.

## Background

The relationship between osteoarthritis (OA) and body weight has been recognized for a long time [1,2]: Body weight is the strongest influenceable predictor of OA. During walking, the body weight is transferred three to six times across the knee joint, showing the great influence body weight has for the risk of developing OA, especially in the knee. The Chingford study, for instance, showed that for every two units increase in body mass index, the odds ratio for developing radiographic knee OA increases by the factor 1.36 [3]. The Framingham study revealed a risk reduction of 50% for women to develop symptomatic OA if the body weight is reduced by about 5 kilograms [4]. Furthermore, prior findings emphasize that not only the incidence of OA can be influenced, but also the progress of symptomatic OA can be reduced if body weight is reduced [5]. Many prior studies focused on the association between obesity and OA in an epidemiological sense. If quality of life (QoL) was assessed, the studies focused on obese people in the general population [6]. Patterns of OA due to obesity were also approached [7], but interestingly less is known about the specific impact of obesity on OA patients regarding quality of life, especially in large samples of unselected primary care patients.

This research deficit is astonishing especially with regard to economic aspects: direct and indirect costs of OA represent a tremendous burden on the health care system [8]. Prior research indicated that these costs could be reduced significantly by weight loss: Coggon et al. estimated that nearly a quarter of surgical interventions might be avoided if obese people reduced their body weight at least by 5 kg or to a normal BMI [9].

Against this background, we assessed the hypotheses that the prevalence of obesity is increased among OA patients and that the disease specific QoL of obese OA patients is significantly reduced compared to OA patients with a normal BMI. Furthermore, we hypothesized that obese patients show an increased use of the health care system compared to control patients with OA and a normal BMI.

## Patients and Methods

The data are extracted from the baseline assessment of the PraxArt project, which is financed by the German Ministry for Education and Research over a period of six years and which aims at improving the quality of life of patients suffering from OA. Data were collected in a large cross-sectional survey including 75 representative General Practitioners (GPs) in the area of Baden-Wuerttemberg and Bavaria, Germany. These GPs created a representative cohort of OA patients to enable a long-time follow-up and the possibility to assess different aspects of QoL and received health care. Some of these analysis have been published elsewhere [10-12].

### Patient inclusion criteria

To be eligible for inclusion, patients had to be adult and diagnosed with arthritis in the hip or knee according to the criteria of the American college of Rheumatology (ACR) [13,14]. In each of the 75 practices, all patients who visited their GP because of complaints related to OA were addressed consecutively until a maximum of 15 patients per practice was reached. After giving their written informed consent, patients received the questionnaire and a return envelope with the postal address of the university. All patients were informed that neither the GP nor the practice team had any possibility to get knowledge of their answers.

### Data collection

Within the project, GPs were asked to note down all patients who would be eligible on a list regardless of whether they agreed to participate or not. This was done in order to enable the comparison between responders and non-responders regarding sociodemographic characteristics later on. Each patient questionnaire was linked to the list with an identification number, so data assessed from patients could be checked by comparing them with the patients' medical files. Sociodemographic data (gender, age, educational level, working situation, family situation) and the following comorbid conditions were retrieved by means of questionnaire: high blood pressure, diabetes, heart insufficiency, coronary vessel disease, elevated cholesterol level (defined as total cholesterol > 200 mg/dl), ulcer or stomach disease, asthma/COPD, renal insufficiency, cancer, and stroke. Radiological severity of OA was graded according to the Kellgren and Lawrence score [15]. Educational level was defined adapted to the German school system and according to the years of education: <= 7 years: 1; 8–10 years: 2; more then 10 years: 3. Where applicable, patients' answers were checked by comparing them with the patients' files. If differences occurred, data from the medical files were used. This procedure was performed to assess accuracy of patients' self reported data later on in the project. The same procedure was performed regarding information about health service utilization, except for the information of complementary and alternative medicine; since these treatments normally do not require a prescription or are not a consequence of referral, they are not recorded in the medical file. Depressive disorders were diagnosed using the depression module of the German form of the Patient Health Questionnaire (PHQ-9) [16]. The PHQ-9 is a self-administered questionnaire that enables diagnosing of Major and Minor Depression according to DSM-IV [17] with a cut-off of 15 points to define depression according to the recommendations of Kroenke et al. [18]. Moreover, the summarized scale score allows assessing the severity of depression. The PHQ-9 has proven to be a valid instrument for those assessments [19,20].

The impact of OA on patients' quality of life was assessed by the GERMAN-AIMS2-SF, which represents a reliable, valid, and comprehensive tool. It provides a comprehensive assessment of QoL while comprising the dimensions physical limitation (divided into upper and lower limb), symptom (reflecting perceived pain), social (reflecting social contacts), affect (reflecting mood), and work (reflecting the ability to work). It has recently been validated in German in a sample of OA patients [21]. As suggested in this study, we separated the physical limitation scale of the AIMS2-SF in upper body limitation and lower body limitation to increase responsiveness.

To assess physical activity (PA), we used the short form of the International Physical Activity Questionnaire (IPAQ) [22]. The IPAQ was developed by an international panel of experts (EUPASS), validated in nine European countries, including Germany, and has frequently been used to assess PA in different European countries [23]. One measure of the volume of activity can be computed by weighting each type of activity with its energy requirements defined in Metabolic Equivalents (METs) to yield a score in MET-minutes, whereby METs are defined as multiples of the resting metabolic rate and a MET-minute is computed by multiplying the MET score of an activity by the minutes it is performed for. MET-minute scores are equivalent to kilocalories for a 60 kilogram person. For vigorous physical activity, the total minutes per week were multiplied with the factor 8, for moderate PA with the factor 4, and for walking with the factor 3.3. The sum of these three products is the MET-min/week. Inactivity is defined as a score below 150 min/week. Individuals are sufficiently active if they perform (1) a minimum of three days of vigorous activity of at least 20 minutes per day, or (2) a minimum of five days with PA of moderate intensity or walking of a least 30 minutes per day, or (3) a minimum of five days of any combination of walking, moderate or vigorous PA accumulating to a total of at least 600 MET-min/week. Individuals are highly active if they perform vigorous PA on a minimum of three days accumulating to at least 1,500 MET-min/week, or seven days of any combination of walking, moderate or vigorous PA accumulating to a total of at least 1,500 MET-min/week. Individuals who neither meet the criteria for inactivity nor sufficient or high activity are insufficiently active. The PA-status (insufficiently active, sufficiently active, and highly active) was defined according to the IPAQ scoring protocol [24]. The categories are adjusted to recommendations of the centres for disease control (CDC) recommendations [23].

### Data were analysed with the SPSS program (version 14.0)

The BMI was calculated on the basis of height and weight, measured after the consultation with the GP. The definitions of the certain groups "normal", "overweight" and "obese" were based on the definition of the World Health Organization (WHO). The study protocol was approved by the ethics committee of the University of Heidelberg previous to the start of the study in January 2005. Inclusion of patients did not start unless there was a written and unrestricted positive vote of the ethics committee. This vote was received in March 2005 (approval number 021/2005).

### Statistical analysis

All data are reported descriptively. Group comparisons between the three BMI groups regarding QoL, health service utilization and physical activity were made by means of analysis of covariance (ANCOVAs). Adjustments were made for covariates such as age, disease duration, the radiological grading according to Kellgren and Lawrence and number of comorbidities where applicable. Dichotomous variables, as for instance comorbidities, were compared by means of Chi-square-test.

## Results

Of the 1,250 distributed questionnaires, 1,021 (81.7%) were returned. In 978 cases, body weight and height were measured by the GPs. 347 (34.0%) of the 1,021 included patients were male and 674 (66.0%) were female. The comparison of patients who returned their questionnaire with the non-responders did not reveal significant differences regarding the following characteristics which could be retrieved form the medical file: sex, age, duration of OA and number of comorbidities as well as number of prescriptions. 278 (80.1%) men and 296 (43.9%) women were married or lived with a partner. Completely retired from work were 233 (67.1%) men and 482 (71.5%) women. Most of the missing data referred to sociodemographic variables and could be completed by means of the patients' medical files. Details about the study sample, separated by BMI, are shown in Table 1. The displayed p-values are the result of group comparisons between the normal weight and the overweight group and between the overweight and the obese group. As can be seen, the groups did not differ regarding age and duration of disease. Both the radiological grading according to Kellgren and the number of comorbidities increased significantly with an increasing BMI. The educational level decreased from normal weighted patients to obese patients.

**Table 1:** Baseline characteristics of study sample

	BMI							
	=<24.9		p*	25–29.9		p*	=>30	

	n	%		n	%		n	%

Total (978)	251	25.7		402	41.1		325	33.1
Female	180	71.7		255	63.4		208	64.0
	Mean	SD		Mean	SD		Mean	SD
Age (in years)	66.9	13.33	0.295	68.09	11.12	0.062	66.36	11.23
Educational level	2.62	1.03	0.039	2.44	0.91	0.033	2.34	0.86
Duration of OA (in years)	13.41	15.18	0.651	13.95	13.27	0.558	13.40	10.79
Amount of comorbidities	1.69	1.283	<0.001	2.19	1.73	<0.001	2.72	1.86
Kellgren score*	2.26	0.68	0.044	2.53	0.92	0.038	2.76	0.77

* by means of ANCOVA, Chi-Square test respectively for comparison normal vs. overweight and overweight vs. obese

Table 2 provides information about the association of patients' comorbidities and the BMI. As can be seen, the prevalence of high blood pressure was significantly higher in the overweight group (compared to normal weight; p < 0.001) and also significantly higher in the obese group than in the overweight group (p = 0.002). Similar findings could be revealed for the prevalence of diabetes since regarding coronary vessel disease the only the differences between the normal weighted patients and the other groups achieved significance but not when we compared over weighted and obese patients. 21 men and 16 women reported about a history of cancer or current cancer disease, significant differences between the groups did not occur.

**Table 2:** Association of comorbidities with obesity (n)

	BMI					Total
	=<24.9	p*	25–29.9	p*	=>30	978 (%)

High blood pressure	95	<0.001	229	0.002	223	547 (55.9%)
Heart insufficiency	37	0.143	78	0.709	67	182 (18.6%)
Coronary vessel disease	15	<0.001	62	0.838	52	129 (13.2%)
Diabetes	15	<0.001	62	<0.001	93	170 (17.4%)
Cholesterol > 200mg/dl	86	0.087	143	0.092	131	360 (36.8%)
COPD/Asthma	13	0.212	32	0.005	50	95 (9.7%)
Renal insufficiency	8	0.139	24	0.421	24	56 (5.7%)
(history of) Ulcer (stomach)	59	0.129	75	0.129	76	210 (21.5%)
(prior) Stroke/TIA/PRIND	6	0.083	23	0.076	12	41 (4.2%)
(history of) cancer	8	0.137	13	0.089	14	36 (3.5%)

* ANCOVA, adjusted for age for comparing normal vs. overweight and overweight vs. obese

Regarding OA specific QoL (Table 3), differences between overweight and normal weight patients were not significant in any dimension, including the PHQ-9 score which was used to assess depression. Significant differences occurred in the lower body scale, the symptom scale, the affect scale and the PHQ-9 score when the BMI surpassed 29.9 m/kg^2 ^in comparison to the overweight as well as to the normal weight group. The upper body scale did not differ between the three groups, a finding which is most likely due to the study sample that consisted only of patients with OA to the knee or hip. Also, there were no significant differences between all groups regarding scores of the social scale, which reflects social network and support, and the work scale. However, it has to be acknowledged that the work scale was only applicable in 263 cases since most of the patients were already retired.

**Table 3:** Impact of BMI on OA related quality of life

	BMI							
	=<24.9		P*	25–29.9		p*	>= 30	

AIMS2-SF scales	Mean	SD		SD	Mean		Mean	SD

Lower body	2.46	1.97	0.409	2.59	2.014	<0.001	3.31	1.89
Upper body	1.47	2.35	0.637	1.56	2.43	0.553	1.46	2.02
Symptom	4.55	2.25	0.158	4.80	2.15	0.029	5.31	2.17
Affect	2.66	1.31	0.073	2.85	1.27	<0.001	3.22	1.42
Social	4.49	1.83	0.173	4.68	1.80	0.173	4.87	1.86
PHQ-9 sum score	14.32	4.59	0.165	14.85	4.44	0.002	16.67	4.93

* by means of ANCOVA, adjusted for age, disease duration, Kellgren and Lawrence-score and number of comorbidities for comparing normal vs. overweight and overweight vs. obese

Table 4 displays the comparison of PA between the three weight groups by means of ANCOVA (adjusted for age, disease duration and comorbidities). As can be seen, PA decreased significantly from patients with normal weight to overweight and to obese patients.

**Table 4:** Physical activity according to IPAQ scoring, separated by BMI

	=<24.9			25–29.9			>= 30	
N	251 (25.7%)		p*	402 (41.1%)		p*	325 (33.1%)	

IPAQ-scoring	Mean	SD		Mean	SD	Mean	SD	

Vigorous activity (min/week)	121.3	(169.1)	0.001	101.2	(155.9)	0.007	70.1	(113.4)
Moderate (min/week)	137.8	(158.2)	0.004	112.8	(147.2)	0.001	94.2	(117.9)
Walking	275.8	(284.8)	0.002	249.9	(271.8)	0.009	242.5	(238.2)
Sitting	2139.2	(879.5)	0.003	2088.1	(855.5)	0.004	2031.9	(977.9)
Total	2674.1	(1959.5)	0.002	2552.0	(1921.5)	0.005	2438.7	(1799.2)
Activity group (%)								
Insufficiently active	126	40.1	0.003	202	50.2	0.008	228	70.1
Sufficiently active	109	43.4	0.067	176	43,8	0.079	89	27.4
Highly active	16	6.3	0.042	24	5.9	0.039	9	2.8

* by means of ANCOVA, adjusted for age, disease duration, Kellgren and Lawrence-score and number of comorbidities for comparing normal vs. overweight and overweight vs. obese

The health service utilization (HSU) patterns of the study sample are displayed in Table 5. In unadjusted analysis, visits to GPs significantly increased with the BMI. Since visits to GPs may often be related to other reasons than OA, we adjusted the ANCOVA for comorbidities (as displayed in Table 5). Interestingly, the significant difference between normal weight and overweight patients faded. However, the difference between obese and normal weight patients remained significant (p = 0.002) even after adjusting for the number of comorbidities. Visits to orthopaedics as well as performed x-rays remained significantly associated with the BMI after adjustment.

**Table 5:** Health service utilization according to BMI

	=<24.9		p*	25–29.9		p*	=>30	
	Mean	SD		Mean	SD		Mean	SD

Contacts to GPs	5.56	9.73	0.059	4.46	5.73	0.051	5.43	7.81
Contacts to Orthopeadics	1.51	2.78	0.049	1.70	2.95	0.001	2.26	4.72
Use of complementary and alternative medicine	0.69	4.99	0.567	0.23	1.47	0.098	0.06	0.66
Use of Physiotherapy	5.36	8.82	0.478	6.97	12.75	0.081	7.78	13.06
Performed x-rays	0.62	3.02	0.003	0.83	3.67	0.002	1.01	4.10

* by means of ANCOVA, adjusted for age, disease duration, Kellgren and Lawrence-score and number of comorbidities for comparing normal vs. overweight and overweight vs. obese

## Discussion

The findings of our study suggest an increased prevalence of overweight and obesity among primary care patients with OA. Furthermore, the burden of OA increased with the BMI and thus confirmed our hypothesis that QoL of OA patients is inversely correlated with the BMI. QoL of patients with OA is mainly determined by pain and physical disability. As our results show, pain as well as physical disability increased with patients' weight. In respect to QoL, patients with OA can be compared to primary care patients in general, as Sach et al. assessed health related quality of life (HRQL) with three different instruments, the EQ-5D, the EQ-VAS and the SF-6D, and also found obesity to be associated with lower HRQL [25].

Bramlage et al. found a prevalence of 37.9% of overweight persons and 19.4% of obese persons among all primary care attendees in Germany [26]. Rates of overweight/obesity increased steadily with the number of comorbid conditions and were highest in patients with diabetes (43.6/36.7%) and hypertension (46.1/31.3%), followed by patients with cardiovascular disorders. With 41.1% overweight and 33.1% obese patients, the prevalence rates we found for patients with OA are significantly higher. Similar results in a cross-sectional study were found by Wannamethee et al. who showed that the prevalence of CV risk factors and morbidity, disability and medication use increased significantly with increasing overweight.

Obese patients were more likely to be referred to a specialist and received significantly more x-rays than non-obese OA patients. Regarding encounters with GPs, the initially significant difference disappeared after adjusting for the number of comorbidities. It can be discussed if this adjustment is appropriate since many of the comorbidities were associated with obesity. However, the focus of this study was OA-related HSU. Nevertheless, the revealed HSU patterns are in line with other findings showing that increase in body weight is associated with increase in medical care costs compared to weight maintenance [27].

The positive effects of PA on the QoL and wellbeing but also on the course as well as on the symptoms of OA has been shown in multiple studies [28]. Especially for patients with OA in the knee, strengthening the musculus quadriceps femoris can reduce pain and slow down the progress of OA most probably mediated by increased stability to the joint [29,30]. Even though a causality can not be assessed due to the cross-sectional design of the study, our results, showing that obese OA patients have a highly significantly reduced physical activity, emphasize the need for life style counselling [31].

Obese patients in our study were significantly more limited in functional disability than non-obese patients. This finding may be due to two different reasons: First of all, the findings regarding perceived pain suggest that these patients simply suffer from more pain that limits functional ability. Secondly, muscle strength, especially the m. quadriceps femoris has been shown to be of great importance for stability of the knee and the incidence, progress and symptoms of OA. As Zoico et al. could show, a high BMI and high body fat were associated with greater probability of functional limitation [32]. The skeletal muscle index (SMI) was the strongest predictor for functional disability of patients (without OA).

Prior research has shown that the prevalence of depression and depressive mood among OA patients is increased compared to the normal population of the same age [20]. Physical limitation (especially to the lower body), pain and social contacts were revealed as most important predictors for a clinically relevant depressive disorder (minor or major depression). Interestingly, overweight was not associated with a higher PHQ-9 score (compared to normal weight) but obesity was. This is an important finding since prior research showed that there is some kind of bidirectional relationship between functional disability and depression: although functional disability can lead to depression, depression has a detrimental effect on physical mobility [33].

The association between obesity and depression has been assessed in a number of studies, including longitudinal studies. Results suggest that obesity predicts later depression [34]. Our data, showing that obese patients have significantly higher PHQ-9 scores, are in line with these findings.

Some weaknesses of our study have to be acknowledged: Our data are not able to assess causality of the association between QoL, physical activity and the BMI. But they confirm the relationship in a large sample of primary care patients and emphasize the influence of obesity on QoL, PA and HSU. Despite the study's weaknesses, to our knowledge, it is the largest study so far assessing the association of BMI, QoL and HSU in primary care patients with OA.

## Conclusion

It has been known for a long time that obesity is the strongest modifiable risk factor for OA and recent research has also shown that the association with the waist circumference is similar [35].

Prior research has shown that GPs' management of overweight and obesity is largely deficient, predominantly due to four interrelated factors: (1) doctors' poor recognition of patients' weight status, (2) doctors' inefficient efforts at intervention, (3) patients' poor acceptance of such interventions, and (4) dissatisfaction with existing life-style modification strategies. Counselling patients to change their lifestyle is a huge challenge, but it has to be the first approach to OA, according to all guidelines. Recent studies suggested that some more intense approaches such as telephone monitoring are able to increase the effect of PA counselling in primary care [11,36]. Evidence based concepts such as the "5A-approach", which originally has been developed for smoking cessation, need to be implemented in the counselling strategy for OA [37]. Our study underlines the need for breaking the vicious circle of increase in body weight, decrease of physical activity, increase in OA related pain, and depression. More research is needed to provide evidence based life style counselling programs to physicians, especially the GP who in most cases is the main care provider for patients with OA.

## Competing interests

The author(s) declare that they have no competing interests.

## Authors' contributions

TR conceived and performed the study and drafted the manuscript. KH performed the data management and statistical calculations. RG, JS and MW participated in the study design and the preparation of the manuscript. All authors read and approved the final manuscript.
